# A novel mitochondrial autophagy and aging-related gene signature for predicting ovarian cancer

**DOI:** 10.3389/fimmu.2025.1594021

**Published:** 2025-06-05

**Authors:** Hongyu Zhang, Qiaoying Jin, Huijuan Cao, Yunyun Wang, Yiling Zhao, Hongwen Zhu, Tiansheng Qin

**Affiliations:** ^1^ Department of Gynecology, The Second Hospital and Clinical Medical School, Lanzhou University, Lanzhou, China; ^2^ Cuiying Biomedical Research Center, The Second Hospital and Clinical Medical School, Lanzhou University, Lanzhou, China

**Keywords:** ovarian cancer, mitochondrial autophagy, cellular senescence, machine learning, prognosis

## Abstract

Ovarian cancer (OC) is a highly malignant gynecologic tumor with a poor prognosis. In recent years, mitochondrial autophagy and aging (MiAg) have been recognized as crucial pathophysiological mechanisms leading to tumorigenesis. However, the expression of MiAg-related genes in OC and their correlation with prognosis remain unclear. In this study, we used multiple machine learning methods to identify 52 MiAg genes that were differentially expressed between OC and normal ovarian tissues. Based on these 52 differentially expressed genes (DEGs), 375 OC patients were classified into two subtypes by consensus clustering analysis. Subsequently, we evaluated the prognostic value of MiAg-related genes in relation to survival in 375 OC patients with complete survival information, and developed a MiAg prognostic score model. By applying Cox and LASSO regression methods, a five-gene signature was constructed, and the 375 OC patients in the TCGA cohort were categorized into low-risk and high-risk group based on the median risk score. Meanwhile, we categorized 174 OC patients from the Gene Expression Omnibus (GEO) database into high- and low-risk groups using the median risk score of the TCGA cohort to validate the MiAg scoring model. Furthermore, we analyzed these data with unifactorial and multifactorial analyses, functional enrichment analysis, gene mutation analysis, immune infiltration, drug susceptibility analysis, cell line analysis, and immunohistochemistry data from the HPA database. In conclusion, the MiAgscore predicted patient survival, and lower MiAgscore values were associated with a better survival advantage. A comprehensive assessment of mitochondrial autophagy and cellular senescence alterations in OC could help advance disease target development and provide more effective personalized treatment strategies for OC patients.

## 1 Introduction

Ovarian cancer (OC) is one of the most common malignant tumors of the female reproductive system, posing a serious threat to women’s health due to its high mortality rate, high recurrence rate, and low 5-year survival rate. The disease often develops asymptomatically in its early stages, and the lack of early and effective screening and diagnostic tools makes diagnosis challenging and frequently delayed (1, 2). The first-line treatments for OC include surgery, chemotherapy, and immunotherapy (3). However, despite significant advances in diagnostic techniques and pharmacological treatments, OC prognosis remains poor, and the low 5-year survival rate combined with chemotherapy resistance continues to present major challenges (4, 5). Therefore, the development of new biodiagnostic markers, therapeutic targets, and drugs is crucial to improve early diagnosis and treatment outcomes for OC patients. Additionally, the establishment of reliable and novel prognostic models could facilitate targeted therapies, thereby improving patient survival and diagnostic accuracy.

Mitochondrial autophagy, which involves the removal of damaged mitochondria to maintain cellular energy supply and stability, plays a crucial role in various diseases when dysregulated—either deficient or excessive (6, 7). It has been shown to significantly impact multiple systemic diseases, including cardiovascular disease, acute pancreatitis, nephropathy, central nervous system disorders, breast cancer, and liver failure (8–13). In ovarian cancer, heightened autophagy in advanced stages enhances cancer cell tolerance to adverse environments, improving tumor cell survival (14). Studies using TCGA and GEO databases indicate that mitochondrial autophagy influences ovarian cancer pathogenesis by modulating the tumor microenvironment (TME), macrophages, stem cells, and drug resistance (15–18). While some suggest autophagy inhibits tumor growth in chemotherapy-resistant ovarian cancer (19), others propose it promotes chemoresistance, highlighting the potential of targeting autophagy to overcome therapeutic resistance (20, 21). However, the contradictory findings and lack of reliable biomarkers for autophagy hinder the clinical application of autophagy-targeted strategies. Thus, further definitive studies on mitochondrial autophagy’s mechanism in ovarian cancer are essential to resolving these discrepancies and identifying novel therapeutic approaches.

Aging significantly impacts the female reproductive system, particularly the ovaries. It influences pregnancy, bone health, cardiovascular health, and cognitive function, highlighting the importance of addressing female aging and its associated health challenges (22). Cellular senescence, a hallmark of aging, involves the irreversible loss of cell replication capacity and morphological degeneration. It serves as a double-edged sword, functioning both as a tumor suppressor and a driver of tumorigenesis in specific contexts (23), playing a crucial role in ovarian cancer development, progression, and treatment. Notably, cellular senescence can modulate chemotherapy efficacy by reducing the required dosage while simultaneously enhancing tumor resistance to apoptosis in ovarian cancer (24, 25). Mitochondrial biogenesis tends to increase with aging, while autophagy decreases. Since the body maintains mitochondrial integrity through autophagy, this process plays a key role in mitigating aging-related damage and disease progression (26). Thus, maintaining mitochondrial health and managing aging changes are closely linked, and preserving normal autophagy levels may help decelerate the aging process.

Recent studies indicate that mitochondrial autophagy and cellular senescence are pivotal in OC. Despite this, no study has systematically utilized mitochondrial autophagy and aging (MiAg)-related genes to develop predictive models for OC prognosis, diagnosis, or immune response. To address this gap, we conducted a comprehensive analysis of MiAg-related genes in OC using public database mining. This analysis compared the expression levels of these genes between normal ovarian tissues and OC tissues, aiming to elucidate their potential significance in OC progression, prognosis, and interactions with the immune microenvironment.

## 2 Materials and methods

### 2.1 Data source

Transcriptomic data and clinical information for this study were obtained from the Xena database (https://xena.ucsc.edu/), including 379 ovarian cancer patients and 88 normal controls. After excluding four samples lacking survival information, the remaining samples were used for subsequent analyses (bioinformatics.com.cn). Additionally, the GSE53963 dataset from the GEO database (https://www.ncbi.nlm.nih.gov/geo/query/acc.cgi?acc=GSE53963) was used as a validation dataset.

### 2.2 Identification of differentially expressed genes related to mitochondrial autophagy and cellular senescence

We downloaded a total of 560 mitochondrial autophagy and cellular senescence-related genes by searching relevant literature (7). To identify differentially expressed genes (MiAg-related DEGs) associated with mitochondrial autophagy and cellular senescence, we performed differential expression analyses using the TCGA and GTEx datasets, comparing gene expression levels between tumor tissues and normal tissues. Gene interaction information was obtained from the Search Tool for the Retrieval of Interacting Genes (STRING, https://cn.string-db.org) database.

### 2.3 Consistent clustering analysis

To investigate the association between MiAg-related DEGs and ovarian cancer subtypes, we analyzed 375 samples from the TCGA-OV cohort by applying a consistent clustering algorithm using the CNSknowall platform (https://cnsknowall.com). The value of the clustering variable k was set between 2 and 10. Heatmaps were generated using the CNSknowall platform, and differences in survival time between subgroups were compared using Kaplan-Meier survival analysis.

### 2.4 Development and validation of a prognostic model for mitochondrial autophagy and cellular senescence-related genes

To assess the prognostic value of mitochondrial autophagy and cellular senescence-related genes, we used Cox regression analysis (R package “survminer” and “survival”) to evaluate the survival status of each gene in relation to patient survival in the TCGA cohort. To ensure a broader initial selection, we set the p-value threshold at 0.1 for screening (27). Candidate genes were then narrowed down using the LASSO Cox regression model (R package “glmnet”) to construct a robust prognostic model. Finally, five key genes and their corresponding coefficients were retained. The riskscore for the 375 samples with complete survival information in the TCGA cohort was calculated using the following formula: Riskscore = (coefficient1 * gene1 expression level) + (coefficient2 * gene2 expression level) +… + (coefficient5 * gene 5 expression level). TCGA ovarian cancer patients were divided into high-risk and low-risk groups based on the median riskscore. Kaplan-Meier analysis was used to compare overall survival (OS) times between the two groups. Additionally, principal component analysis (PCA) (R package “dplyr”) and receiver operating characteristic (ROC) curve analysis (R package “timeROC”) were performed to assess the model’s predictive accuracy. For validation, we used the GSE53963 ovarian cancer dataset from the GEO database, applying the median risk score from the TCGA dataset as the cutoff value to categorize patients into high-risk and low-risk groups.

### 2.5 Independent prognostic analysis of risk scores

To determine whether riskscores serve as an independent prognostic factor, we extracted clinical information from the TCGA and GEO cohorts and performed univariate and multivariate Cox regression analyses. Furthermore, to evaluate the clinical applicability of the risk model, we constructed nomograms using R packages (“rms,” “survival,” and “regplot”) and validated them with calibration curves.

### 2.6 Functional enrichment analysis of DEGs in low and high risk groups

Ovarian cancer patients in the TCGA cohort were categorized into high-risk and low-risk groups based on the median risk score. Subsequently, differentially expressed genes between the two groups were screened according to the criteria of |log2FC| ≥ 1 and Pavle < 0.05. Based on these identified DEGs, GO and KEGG functional enrichment analyses were performed to explore their biological significance. In addition, we downloaded somatic mutation data from the TCGA website (https://portal.gdc.cancer.gov/) and compared the differences in the top 30 most frequently mutated genes between the high-risk and low-risk groups using R packages (maftools).

### 2.7 Correlation analysis of mitochondrial autophagy and cellular senescence-related gene model with immune checkpoints and immune cell infiltration

To investigate the effect of the model on immunotherapy response, we analyzed the differences in immune checkpoint expression between the high-risk and low-risk groups. Additionally, we downloaded the immune score data from the cancer immunome atlas (TCIA) database (https://tcia.at/). To evaluate the immune cell infiltration in ovarian cancer, we used the single-sample gene set enrichment analysis (ssGSEA) algorithm to generate a heatmap illustrating 28 immune cell types through the biocloudservice tool (http://www.biocloudservice.com/). Furthermore, we assessed the immune infiltration levels and immune function scores of 29 immune-related pathways between the high-risk and low-risk groups using R software packages (“GSVA” and “BiocManager”).

### 2.8 Immune checkpoints analysis and drug sensitivity analysis

To explore more deeply the relationship between the immune environment and tumor immune escape mechanisms, we analyzed the expression levels of 19 common immune checkpoints and their correlations in a subgroup of patients with high and low MiAg scores. To explore the relationship between the expression levels of mitochondrial autophagy and cellular senescence-related genes and chemotherapeutic response, we selected 10 commonly used chemotherapeutic agents and analyzed the differences in half-maximal inhibitory concentration (IC50) values between the two risk score groups using the R software package (26).

### 2.9 CCLE and HPA database analysis

To facilitate subsequent experimental validation through cellular assays and immunohistochemical analyses, we used the Cancer Cell Line Encyclopedia (CCLE, https://sites.broadinstitute.org/ccle) database to compare gene expression differences across ovarian cancer cell lines between the high-risk and low-risk groups. Additionally, we used the Human Protein Atlas (HPA, https://www.proteinatlas.org/) database to further validate the expression patterns of the five prognostic genes in ovarian cancer patients.

## 3 Results

**Figure 1:**
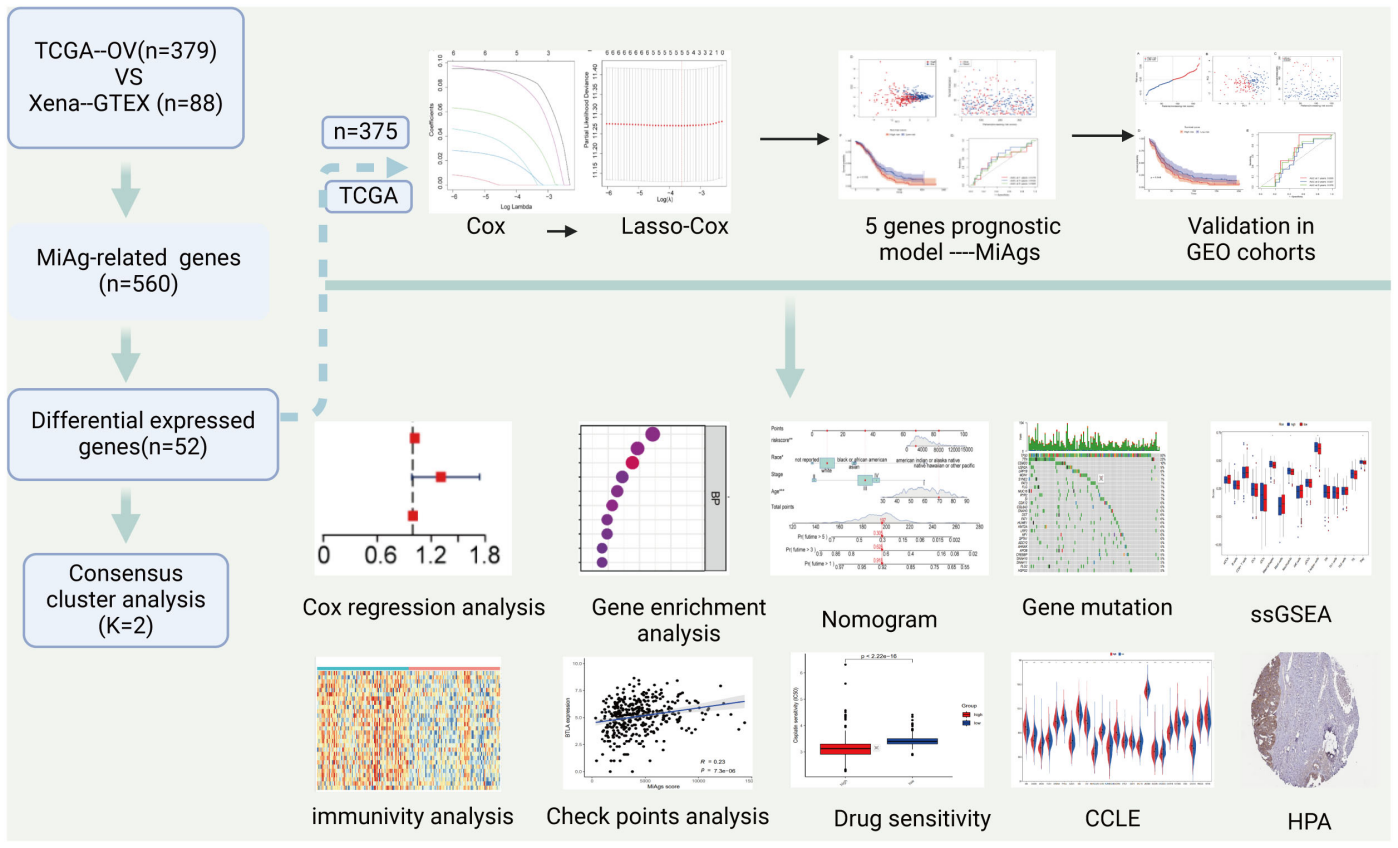
Provides a flowchart of the entire workflow and study design. Our findings contribute to a better understanding of the role of the MiAg gene in OC and provide insights for identifying novel prognostic markers and therapeutic targets in OC. (Created in BioRender).

**Figure 1** presents a flowchart outlining the entire study workflow and design. Our findings provide novel insights into the role of MiAg genes in OC, contributing to the identification of potential prognostic markers and therapeutic targets.

### 3.1 Identification of DEGS between normal and tumor tissues

**Figure 2:**
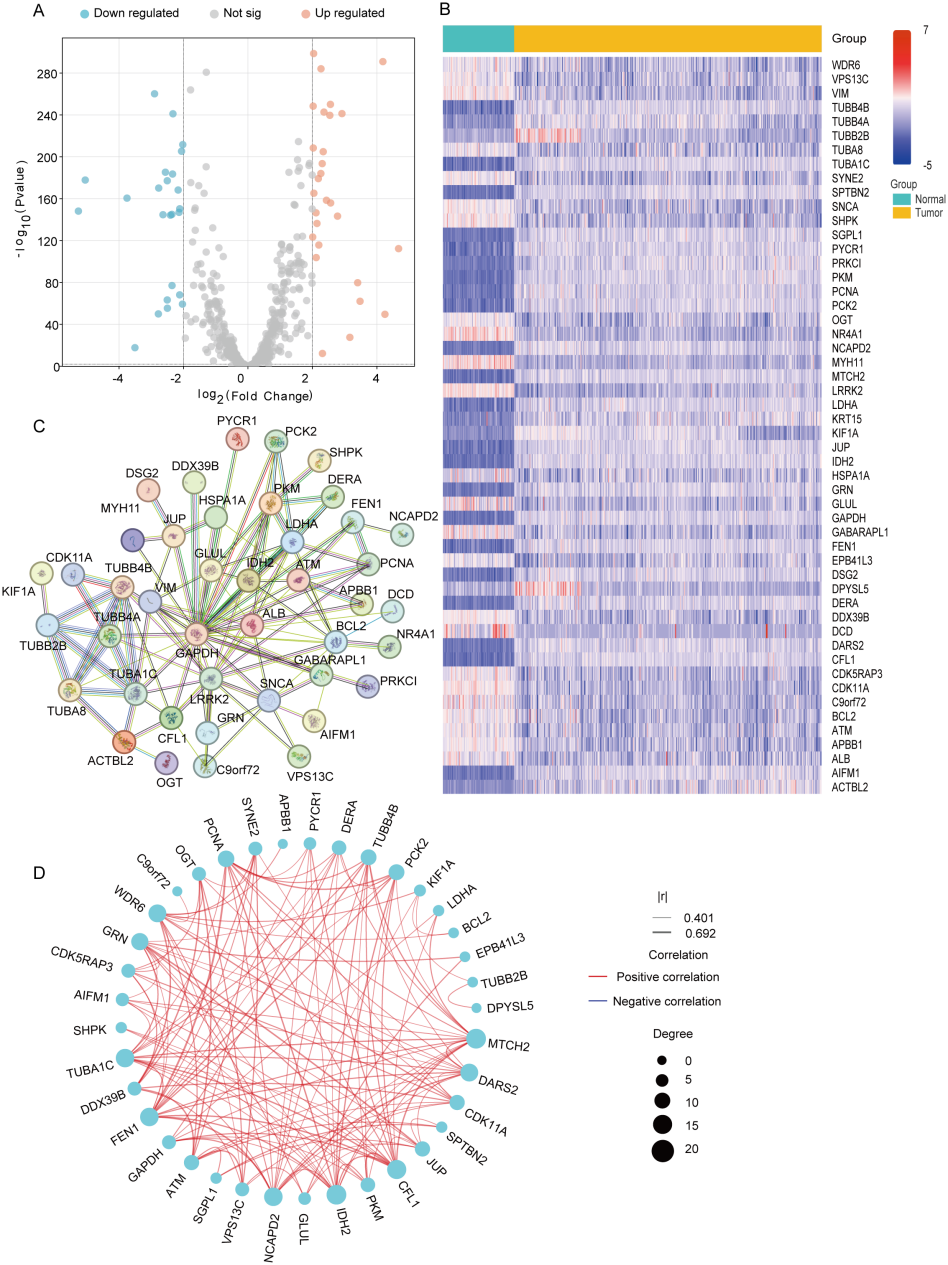
Expression and interaction of 52 mitochondrial autophagy and cellular senescence related genes. **(A)** Volcano plot of MiAg-related DEGs between ovarian cancer and normal. **(B)** Heatmap of mitochondrial autophagy and cellular senescence differential genes between normal tissues (blue) and tumor tissues (yellow) (Blue: low expression level; Red: high expression level; |log2FC |>2, P<0.01). **(C)** PPI network showing the interaction of mitochondrial autophagy and cellular senescence differential genes. **(D)** Correlation network diagram of mitochondrial autophagy and cellular senescence differential genes (Red line: positive correlation; Blue line: negative correlation; The depth of the color reflects the strength of the correlation).

Using the Xena database, we obtained transcriptomic data from 379 ovarian cancer tumor tissue samples and 88 normal ovarian tissue samples. We then analyzed the expression levels of 560 MiAg-related genes in normal and tumor tissues. DEGs were identified using the criteria |log2FC| > 2 and p < 0.01, resulting in 52 significant DEGs (**Figure 2A**). Among them, 24 genes were downregulated in tumor tissues, while 28 genes were upregulated. The expression profiles of these 52 DEGs in normal versus tumor tissues were visualized using a heatmap (**Figure 2B**), where blue represents low expression levels and red represents high expression levels. To further explore potential functional interactions among these DEGs, we performed protein-protein interaction (PPI) analysis using the STRING database. The PPI network revealed 10 hub genes with the highest connectivity, namely TUBB4B, TUBA1C, TUBB2B, GAPDH, LRRK2, ALB, MAPT, TUBB4A, TUBA8, and GJA1. Each of these hub genes exhibited interactions with more than 10 other genes, suggesting their crucial roles in OC pathogenesis (**Figure 2C**). Additionally, we constructed a correlation network graph to illustrate the relationships among the 52 DEGs (**Figure 2D**), where red indicates a positive correlation between genes.

### 3.2 Tumor classification based on DEGS

**Figure 3:**
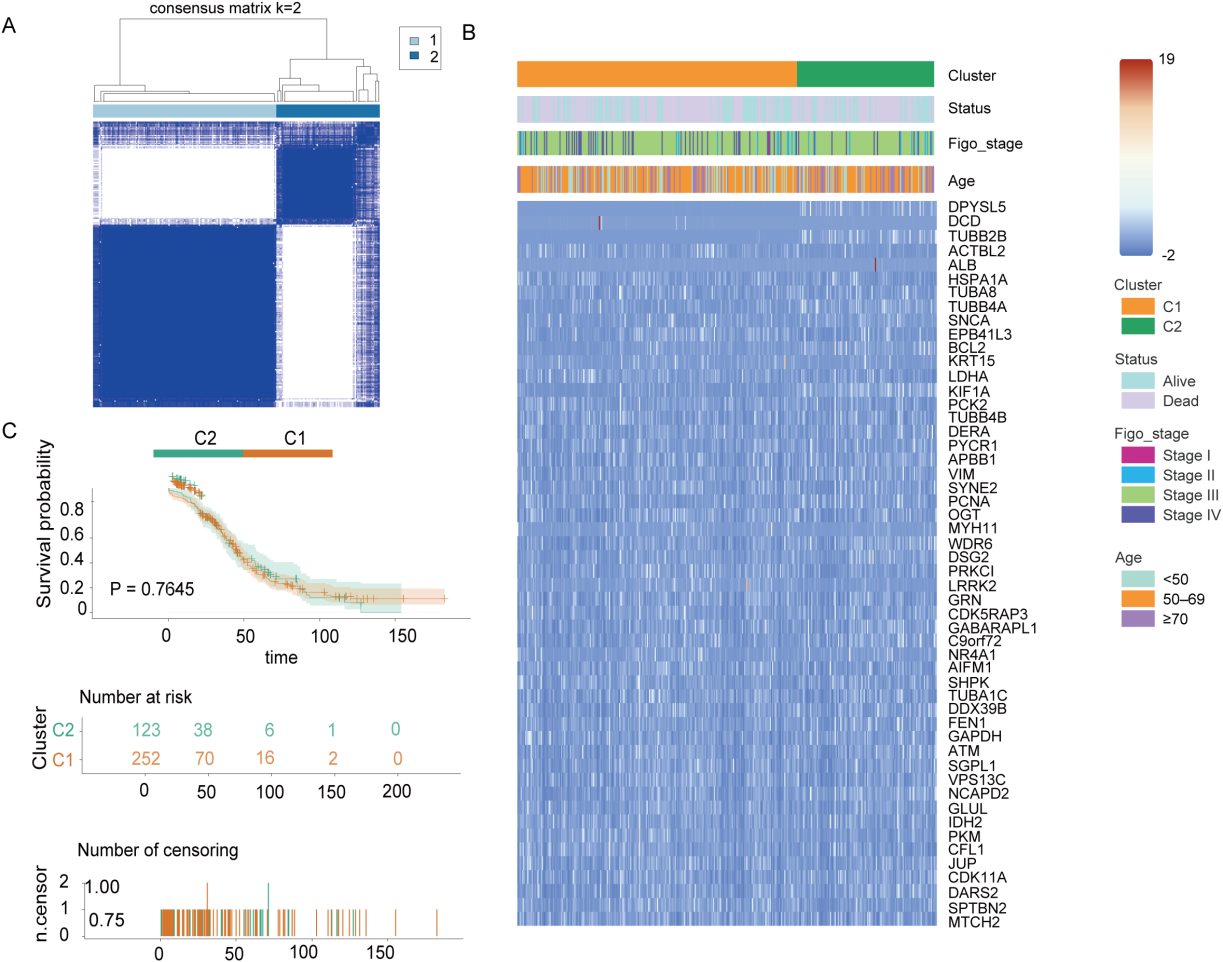
Tumor classification based on mitochondrial autophagy and cellular senescence-associated DEGS Figure. **(A)** 375 OC patients were classified into two clusters according to the consensus clustering matrix (k = 2). **(B)** Heatmap and clinicopathological characteristics of the two clusters classified by these DEGs. **(C)** Kaplan-Meier OS curves for the two cluster.

To investigate the role of the 52 DEGs associated with mitochondrial autophagy and cellular senescence in OC, we conducted a consensus clustering analysis using 375 OC patient samples with complete survival and clinical data from The Cancer Genome Atlas (TCGA) database. We systematically evaluated clustering solutions for k = 2 to k = 10 and determined that k = 2 provided the most stable and biologically meaningful classification, maximizing intra-group similarity while minimizing inter-group differences. This analysis stratified OC patients into two distinct molecular subtypes based on DEGs expression profiles (**Figure 3A**). To further characterize these subtypes, we visualized heatmaps that integrated gene expression patterns with clinical parameters, including tumor grade (FIGO stage), patient age (<50, 50–69, ≥70 years), and survival status (alive or dead) (**Figure 3C**). However, we observed minimal differences in clinical characteristics across the two subtypes. Additionally, to assess the prognostic significance of these subtypes, we conducted Kaplan-Meier survival analysis to compare overall survival (OS) times across the two groups. The results indicated no statistically significant difference in OS among the subtypes (p = 0.76, **Figure 3B**), suggesting that while the DEGs effectively classified OC patients into distinct molecular subtypes, these classifications did not correlate with survival outcomes. This phenomenon may be related to the low proportion of long-surviving patients in the sample-only 77 of those who survived more than 60 months, or less than 25%-which may have limited the statistical power of the survival analysis.

### 3.3 Development of prognostic genetic models for the TCGA cohort

**Figure 4:**
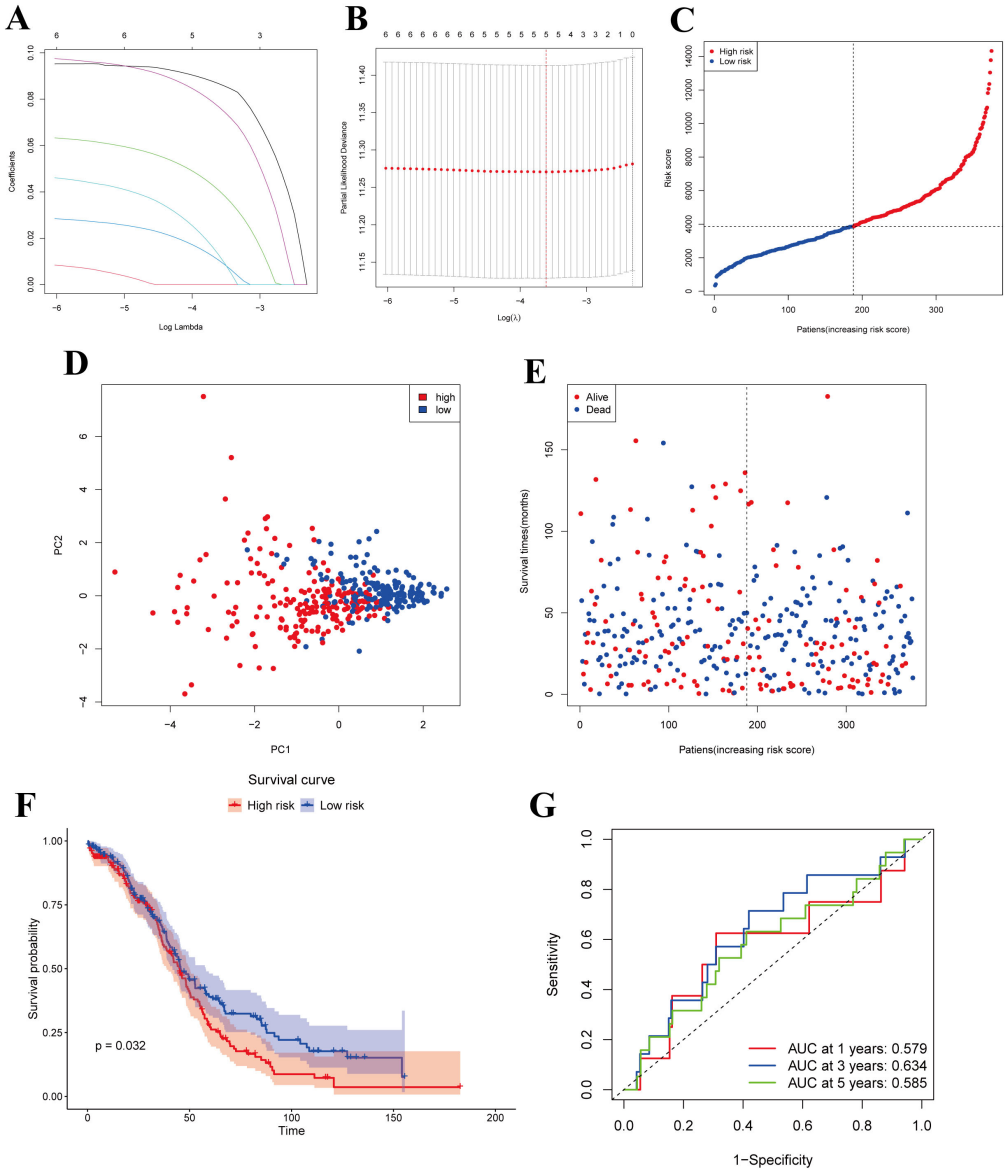
Construction of risk models in the TCGA cohort. **(A)** LASSO regression for 5 OS-related genes. **(B)** Cross-validation to adjust parameter selection in LASSO regression. **(C)** Distribution of patients based on risk scores. **(D)** PCA plots of survival of OC patients based on risk scores. **(E)** Survival status for each patient. **(F)** Kaplan-Meier OS curves for patients in the high-risk group and patients in the low-risk group. **(G)** ROC curves demonstrating the predictive efficiency of the risk scores.

We obtained a total of 375 OC samples with complete survival and clinical data from TCGA database. To identify MiAg-related genes associated with survival, we conducted univariate Cox regression analysis on 52 DEGs associated with MiAg. Genes with P < 0.1 were retained for further analysis, all of which exhibited hazard ratios (HR) > 1, indicating their association with increased risk in OC patients. To refine our selection, we applied Least Absolute Shrinkage and Selection Operator (LASSO) regression, which identified five independent prognostic genes: JUP, NR4A1, GABARAPL1, PRKCI, and EPB41L3 (**Figures 4A, B**). Based on these genes, we developed a novel prognostic signature, the MiAGs score, to evaluate clinical survival outcomes in OC patients. The risk score was calculated using the following formula: Risk score=(0.086692754×JUP expression) + (0.040674607×NR4A1expression) + (0.010531697×GABARAPL1expression) + (0.012828561×PRKCI expression) + (0.076699495×EPB41L3 expression). Patients were then stratified into low-risk and high-risk groups based on the median riskscore (**Figure 4C**). Principal Component Analysis (PCA) demonstrated distinct separation between high-risk and low-risk groups (**Figure 4D**), highlighting the robustness of the MiAGs score in classification. Additionally, patients in the high-risk group exhibited higher mortality rates and shorter survival times compared to those in the low-risk group (**Figure 4E**). Kaplan-Meier survival analysis demonstrated that high-risk patients had significantly shorter overall survival than those in the low-risk group (P = 0.032, **Figure 4F**), corroborating the prognostic value of the MiAgs score. Furthermore, we performed time-dependent ROC analysis to evaluate the predictive accuracy of the model. The Area Under the Curve (AUC) values were 0.579 at 1 year, 0.634 at 3 years, and 0.585 at 5 years (**Figure 4G**), suggesting moderate predictive capability for medium-term survival outcomes. In conclusion, the MiAGs score, derived from five mitochondrial autophagy-related genes, effectively stratifies OC patients into distinct risk groups with significant differences in survival outcomes. This prognostic signature holds clinical potential for predicting patient prognosis and guiding personalized treatment strategies in ovarian cancer management.

### 3.4 External validation of the risk model

**Figure 5:**
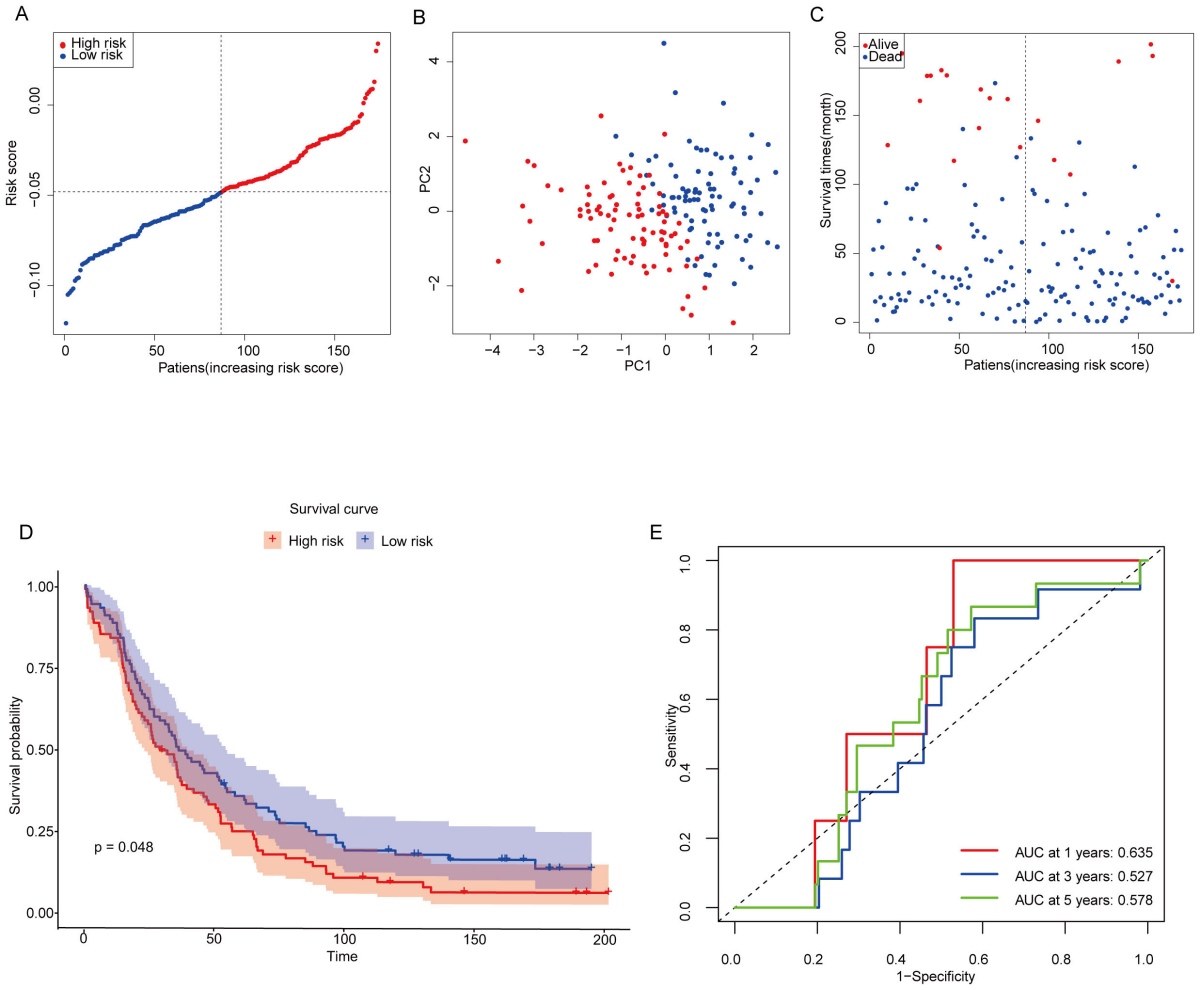
Validation of constructing risk models in the GEO cohort. **(A)** Distribution of patients based on risk scores in the GSE53963 cohorts. **(B)** PCA plots of survival of OC patients based on risk scores. **(C)** Survival status for each patient. **(D)** Kaplan-Meier OS curves for patients in the high-risk group and patients in the low-risk group. **(E)** ROC curves showing the predictive efficiency of the risk scores.

The 174 OC patients from the GEO cohort (GSE53963) were included as the validation set. Patients were categorized into low-risk and high-risk groups based on the median risk score derived from the TCGA cohort. Specifically, 87 patients were assigned to the low-risk group, while the remaining 87 patients were classified into the high-risk group (**Figure 5A**). To further evaluate the distinction between these groups, PCA analysis was performed to assess the separation between the low-risk and high-risk groups within the GEO cohort. The results clearly demonstrated that patients were effectively divided into two distinct clusters based on their risk scores (**Figure 5B**). Patients in the low-risk subgroup had a significantly higher probability of long-term survival than those in the high-risk group (**Figure 5C**). To validate this observation, Kaplan-Meier survival analysis was conducted to assess the survival differences between the low-risk and high-risk groups. Notably, Kaplan-Meier analysis showed that OC samples with lower MiAgs scores were significantly associated with good outcomes (P = 0.048) (**Figure 5D**). This finding underscores the prognostic power of the risk score model in distinguishing between patients with favorable and unfavorable outcomes, thereby supporting its potential for clinical application. The areas under the ROC curves were 0.635 for 1-year, 0.527 for 3-year, and 0.578 for 5-year survival. These AUC values indicate that the model has moderate predictive accuracy (**Figure 5E**), particularly at the 1-year time point. While the predictive power diminishes slightly at longer time points, the overall results demonstrate the model’s potential to differentiate between high-risk and low-risk patients, with its predictive strength being most pronounced in the short term.

### 3.5 Independent prognostic value of risk models

**Figure 6:**
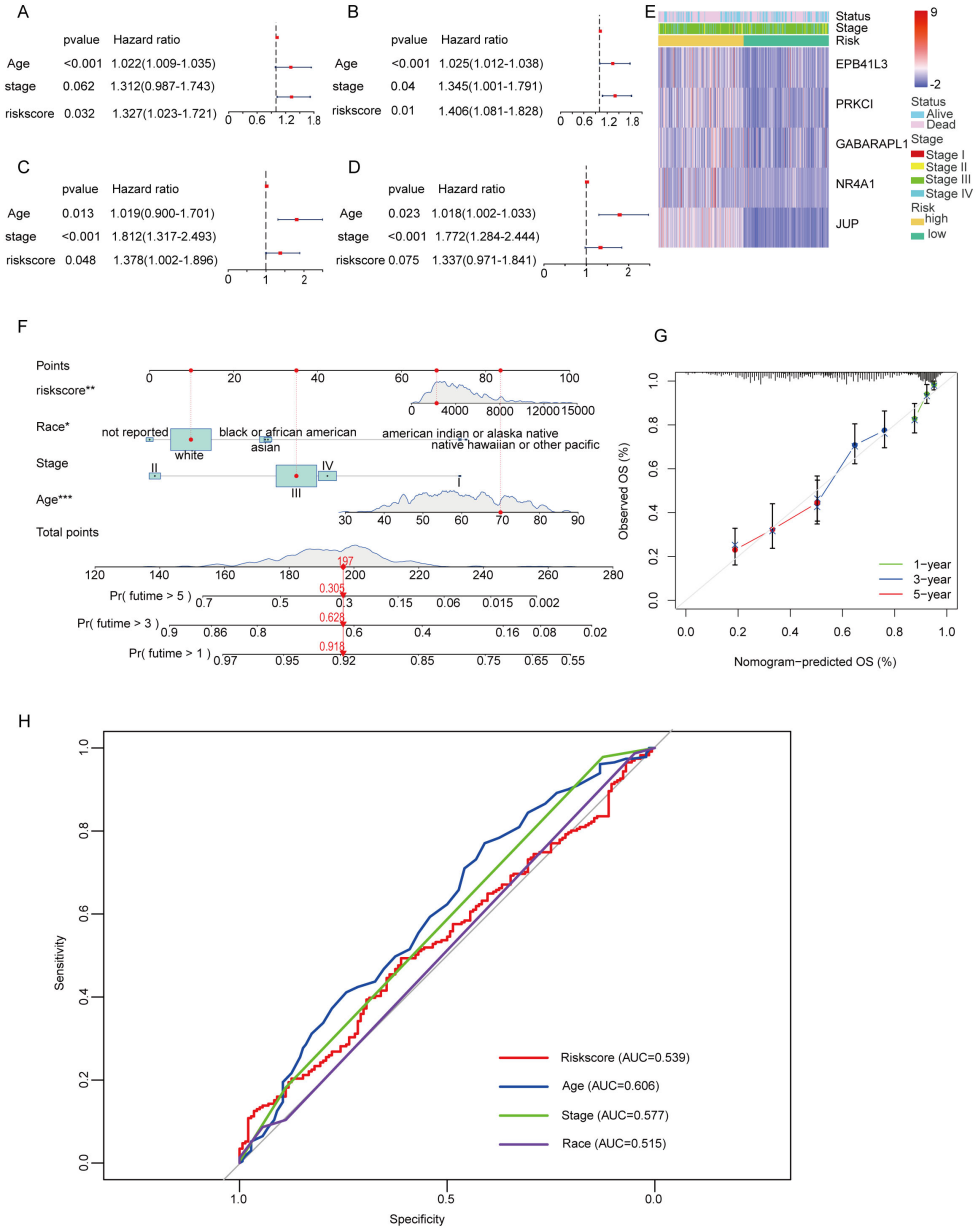
Univariate and multivariate Cox regression analyses for the risk score and construction of a nomogram. **(A)** Univariate analysis for the TCGA cohort. **(B)** Multivariate analysis for the TCGA cohort. **(C)** Univariate analysis for the GEO cohort. **(D)** Multivariate analysis for the GEO cohort. **(E)** An heatmap displays the variation in expression MiAg genes and the clinicopathological differences between the low- and highscore groups. **(F)** Nomogram for predicting the probability of 1-, 3-, and 5-year overall survival. **(G)** Calibration curve of the nomogram. **(H)** The AUC curve for the Nomogram model.

In the TCGA training cohort, univariate Cox regression analysis was performed to identify potential prognostic factors for overall survival in OC patients. The results indicated that age (hazard ratio [HR] = 1.022, 95% confidence interval [CI] = 1.009-1.035, p < 0.001) and risk score (HR = 1.327, 95% CI = 1.023-1.721, p =0.032) were significantly associated with overall survival (**Figure 6A**). These findings suggest that both age and the risk score derived from the prognostic model are important predictors of survival outcomes in OC patients. To further elucidate the independent prognostic significance of the identified factors, multivariate Cox regression analysis was conducted. The results revealed that age (HR = 1.025, 95% CI = 1.012-1.038, p < 0.001) and risk score (HR = 1.406, 95% CI = 1.081-1.828, p =0.01) remained significant prognostic factors in the multivariate model (**Figure 6B**). These findings highlight the importance of age and risk score as independent predictors of survival. Interestingly, although the GEO dataset showed that age and stage were independent predictors of survival (**Figures 6C, D**), differences in stage and risk scores were observed between TCGA and GEO. By analyzing the two datasets, this variation may be due to differences in sample size, methods of data collection, sequencing platforms, and methods of raw data processing. In addition, we analyzed the heatmap of the expression distribution of five independent prognostic MiAg genes in the low-risk and high-risk groups along with their clinical characteristics, and the results showed that the expression levels of JUP, NR4A1, GABARAPL1, PRKCI, and EPB41L3 were higher in OC samples with high MiAgs scores (**Figure 6E**), further supporting their potential role in disease progression.

To facilitate the clinical application of the MiAg score, we developed a nomogram based on the significant prognostic factors identified in the TCGA cohort, including age, disease stage, and risk score. The nomogram provides a visual tool for predicting the 1-, 3-, and 5-year overall survival probabilities for both high-risk and low-risk groups (**Figure 6F**). Notably, in this nomogram, the risk score and age are the primary contributors to the prediction of survival probability, reflecting their significant impact on patient outcomes. Moreover, race/ethnicity also plays a role in predicting overall survival (OS), highlighting the importance of considering demographic factors in prognostic models. To assess the accuracy and reliability of the nomogram, we generated calibration curves comparing the predicted overall survival probabilities with the actual observed survival outcomes (**Figure 6G**). The calibration curves demonstrate that the nomogram-predicted OS closely aligns with the actual OS, indicating high level of reliability and accuracy. To provide further clear evidence, we provide a nomogram of the ROC curve from and evaluate the predictive performance of the model (**Figure 6H**). Specifically, the calibration plots show minimal deviation from the ideal 45-degree line, suggesting that the model performs well across different time points. This consistency between predicted and observed survival probabilities underscores the nomogram’s potential for clinical application, providing clinicians with a valuable tool for estimating individual patient survival probabilities.

### 3.6 Risk model-based functional enrichment analysis

**Figure 7:**
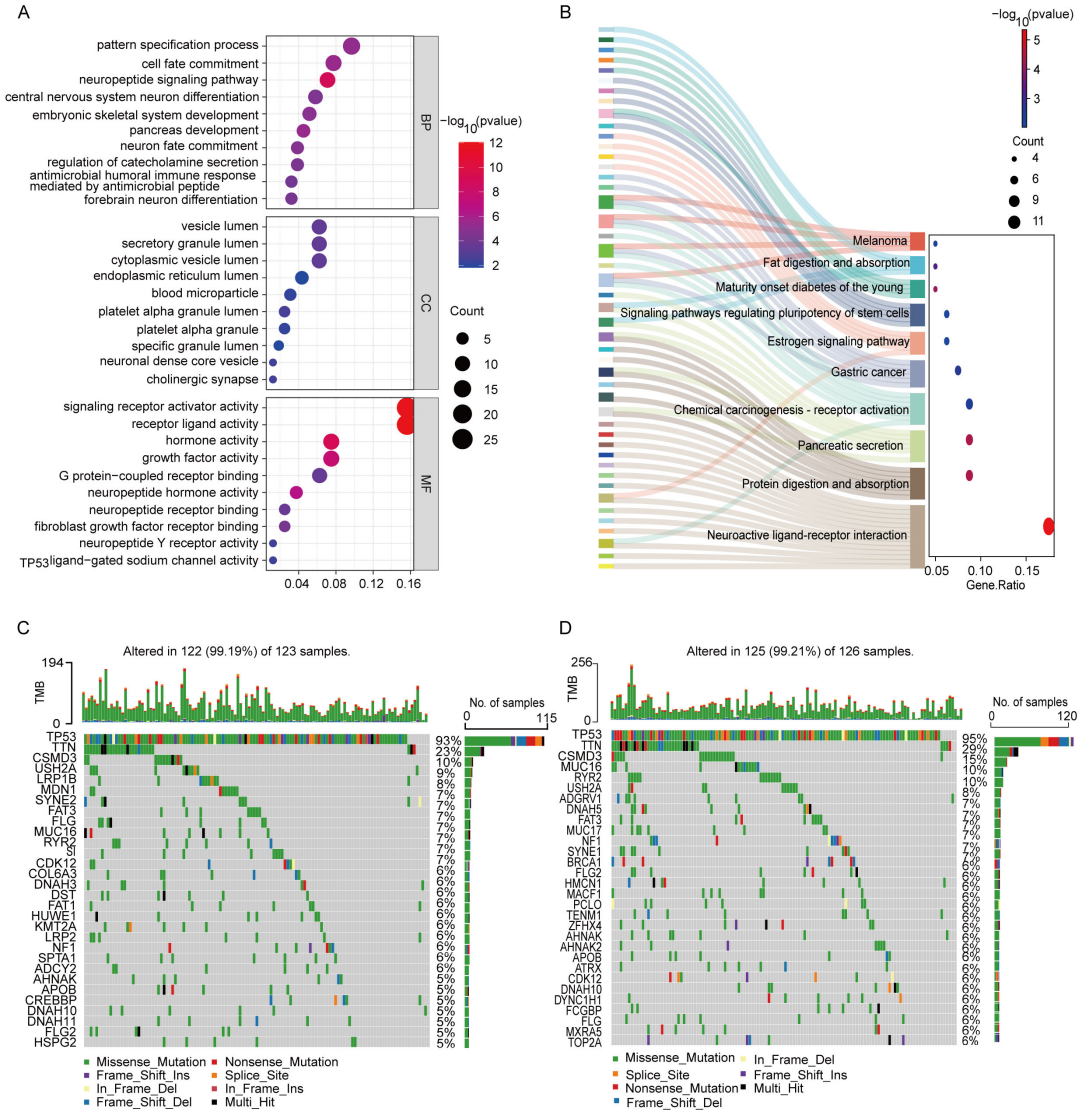
Functional analysis based on the DEGs and mutational load between the two-risk groups in the TCGA cohort. **(A)** GO analysis based on DEG between two risk groups in the TCGA cohort. **(B)** KEGG enrichment analysis. **(C)** Waterfall plot of somatic mutations in the high-risk group. **(D)** Waterfall plot of somatic mutations in the low-risk group.

To further explore the differences in gene functions and pathways between subgroups categorized by risk models, we analyzed the TCGA data of the high- and low-risk groups using differential expression criteria of P < 0.05 and |log2FC| ≥ 1.5, and a total of 230 DEGs were identified. Among them, 38 genes were upregulated in the high-risk group, while the remaining 192 genes were downregulated. Gene Ontology (GO) analysis was performed to elucidate the biological functions and processes associated with the selected prognostic genes. The analysis revealed significant enrichment in several key biological processes, cellular components, and molecular functions (**Figure 7A**). Specifically, the enriched biological processes (BP) included “pattern specification process,” “cell fate commitment,” “neuropeptide signaling pathway,” and “central nervous system neuron differentiation,” all of which are crucial for cell development, immune response, and neuronal function, implying their potential involvement in ovarian cancer progression and therapeutic response. The cellular components (CC) were enriched in “vesicle lumen,” “secretory granule lumen,” and “neuronal dense core vesicle” indicating their involvement in vesicle formation and secretion, which play essential roles in cellular communication, protein trafficking, and immune response. The molecular functions (MF) were enriched in “signaling receptor activator activity,” “receptor ligand activity,” “hormone activity,” and “G protein-coupled receptor binding”. These functions suggest roles in signal transduction and receptor-mediated signaling, which are critical for modulating cellular responses, intercellular signaling, and immune regulation in ovarian cancer.

KEGG pathway analysis was conducted to identify the signaling pathways associated with prognostic genes (**Figure 7B**). The analysis revealed significant enrichment in several pathways, including “Neuroactive ligand-receptor interaction,” “Protein digestion and absorption,” “Pancreatic secretion,” “Chemical carcinogenesis-receptor activation,” “Estrogen signaling pathway,” and “Gastric cancer.” These pathways highlight the genes’ involvement in neuroactive signaling, digestive processes, metabolism, hormonal responses, and cancer-related pathways, suggesting their potential roles in tumor progression and disease regulation. Notably, the identification of these pathways indicates their potential as therapeutic targets and biomarkers, which may provide novel insights into precision medicine approaches for ovarian cancer.

We conducted an analysis to explore the differences in somatic mutation profiles between high-risk and low-risk OC patients (**Figures 7C, D**). Focusing on the top 30 genes, we observed significant variations in the distribution and frequency of mutated genes, despite similar overall mutation rates (99.19% in the high-risk group versus *vs*. 99.21% in the low-risk group). Notably, 13 genes were shared between the two groups, with TP53 (93% and 95%), TTN (23% and 29%), and CSMD3 (10% and 15%) being the most frequently mutated. Beyond these shared genes, the high-risk group exhibited elevated mutation frequencies in USH2A and FLG, which may contribute to the more aggressive nature of the disease. Conversely, genes such as RYR2, NF1, AHNAK, APOB, MUC16, FLG2, and DNAH10 were less mutated in the high-risk group, suggesting potential protective roles or alternative disease mechanisms. These findings underscore the importance of considering mutational profiles in risk stratification and the development of personalized treatment strategies for OC patients, highlighting potential targets for precision oncology.

### 3.7 Comparison of immunoreactivity between subgroups

**Figure 8:**
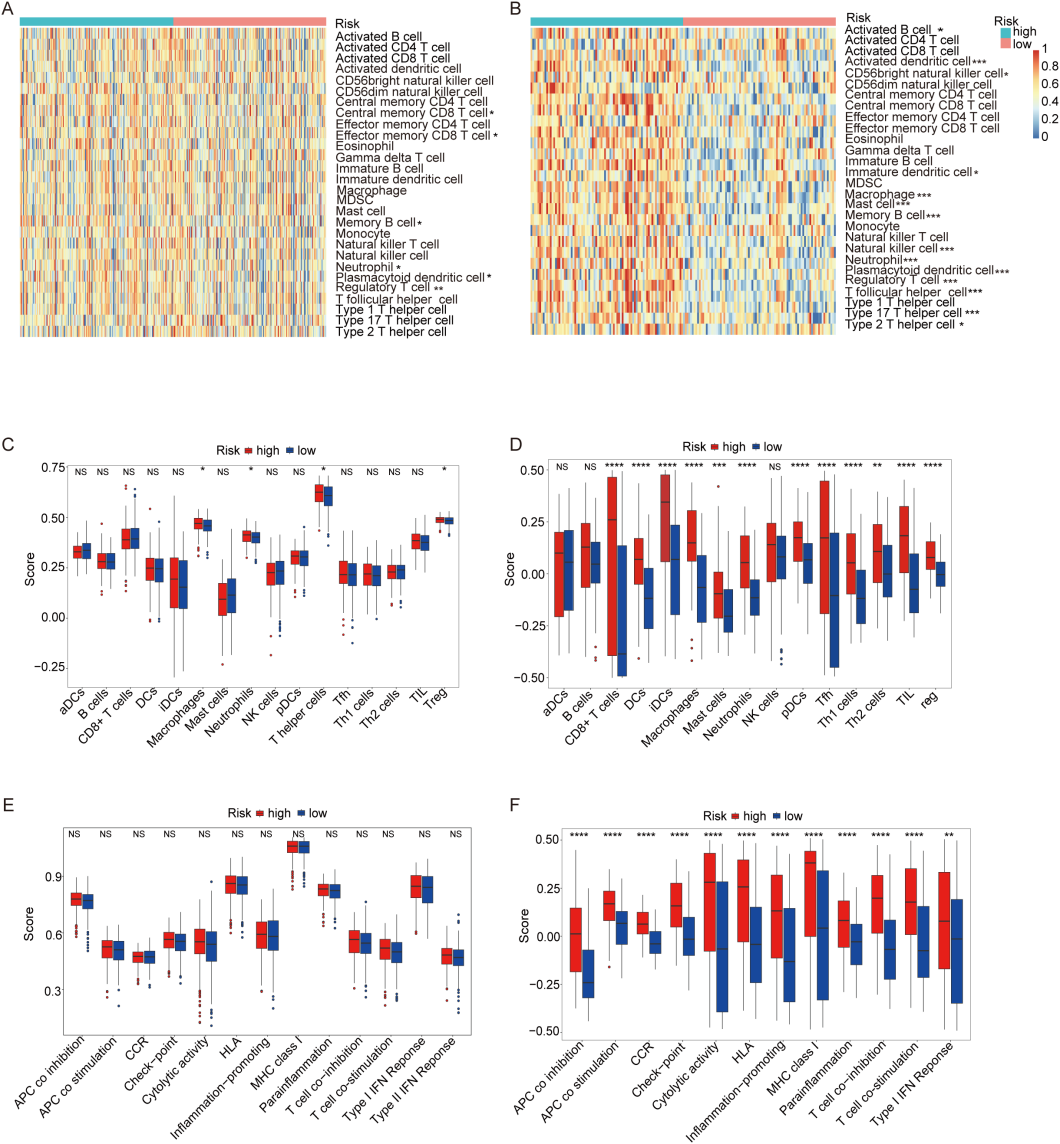
ssGSEA score comparison of immune cells and immune pathways. **(A)** Heatmap of 28 immune cells between low-risk (blue) and high-risk (red) groups in the TCGA cohort. **(B)** Heatmap of 28 immune cells between two groups in the GEO cohort. **(C)** Comparison of enrichment scores of 16 immune cells between two groups in the TCGA cohort. **(D)** Comparison of enrichment scores for 16 immune cells and 13 immune-related pathways between two groups in the GEO cohort. **(E)** Comparison of enrichment scores of 13 immune-related pathways between two groups in the TCGA cohort. **(F)** Comparison of enrichment scores for 13 immune-related pathways between two groups in the GEO cohort.(p-values are shown as: ns not significant. *P < 0.05; **P < 0.01; ***P < 0.001; ****P < 0.0001).

**Figure 8A** illustrates the immune cell infiltration profiles of 28 distinct cell types in the TCGA ovarian cancer cohort, comparing high-risk and low-risk patient groups. The high-risk group exhibited significantly elevated infiltration of Central memory CD8 T cell and effector memory CD8^+^ T cells, suggesting enhanced adaptive immunity and potential anti-tumor activity. This group also showed higher neutrophil infiltration, potentially supporting anti-tumor responses, and increased memory B cell levels, indicative of humoral immune activation. Conversely, the high-risk group displayed a predominant infiltration of regulatory T cells (Tregs) and plasmacytoid dendritic cells (pDCs), indicating an immunosuppressive microenvironment that may contribute to tumor progression. These contrasting immune landscapes underscore the prognostic relevance of immune cell dynamics in OC. In the GEO cohort (**Figure 8B**), Central memory CD8 T cell and effector memory CD8 T cells did not show statistically significant differences between risk groups, contrasting with the TCGA results. However, memory B cells remained more abundant in the high-risk group, consistently aligning with TCGA findings. Similarly, Tregs and pDCs were enriched in the high-risk group, further reinforcing the association between immunosuppression and poor prognosis observed in both cohorts.

**Figure 8C** presents a heatmap analysis of 16 distinct immune cell types within the TCGA cohort of ovarian cancer patients, highlighting differences between high-risk and low-risk groups. The high-risk group exhibited significantly higher infiltration levels of macrophages, neutrophils, T helper cells, and Tregs, suggesting a more immunosuppressive tumor microenvironment. The elevated presence of these immunosuppressive cells in the high-risk group may contribute to a poorer prognosis by suppressing the anti-tumor immune response. **Figure 8D** validates these findings in the GEO cohort (**Figure 8D**), providing additional evidence for the observed immune infiltration patterns. Consistently, the high-risk group in the GEO cohort also exhibits higher infiltration of macrophages, neutrophils, and Tregs compared to the low-risk group, reinforcing the link between immunosuppression and adverse outcomes. Interestingly, T helper cells were not detected in the GEO cohort, suggesting potential variability in immune cell infiltration patterns across different patient populations.

**Figure 8E** illustrates a heatmap analysis displaying the levels of 13 immune features within the TCGA cohort of OC patients, comparing high-risk and low-risk groups. Notably, all 13 immune none of these differences reached statistical significance, suggesting that while there are observable variations in immune feature levels between the two risk groups, these differences may not be sufficiently robust to establish statistical significance without further validation. **Figure 8F** presents a similar analysis of immune feature levels in the GEO validation cohort (**Figure 8F**), focusing on the same 13 immune features as in the TCGA cohort, except for T cell co-inhibition, which was absent in the GEO data. The analysis reveals that 12 out of the 13 immune features displayed significantly higher levels in the high-risk group compared to the low-risk group. These findings suggest that the high-risk group may possess a distinct immune profile characterized by elevated levels of several immune features, which could indicate a more complex or active immune environment in these patients. This highlights the importance of considering immune feature profiles in risk stratification and personalized treatment strategies for OC patients.

### 3.8 Immune checkpoints analysis

**Figure 9:**
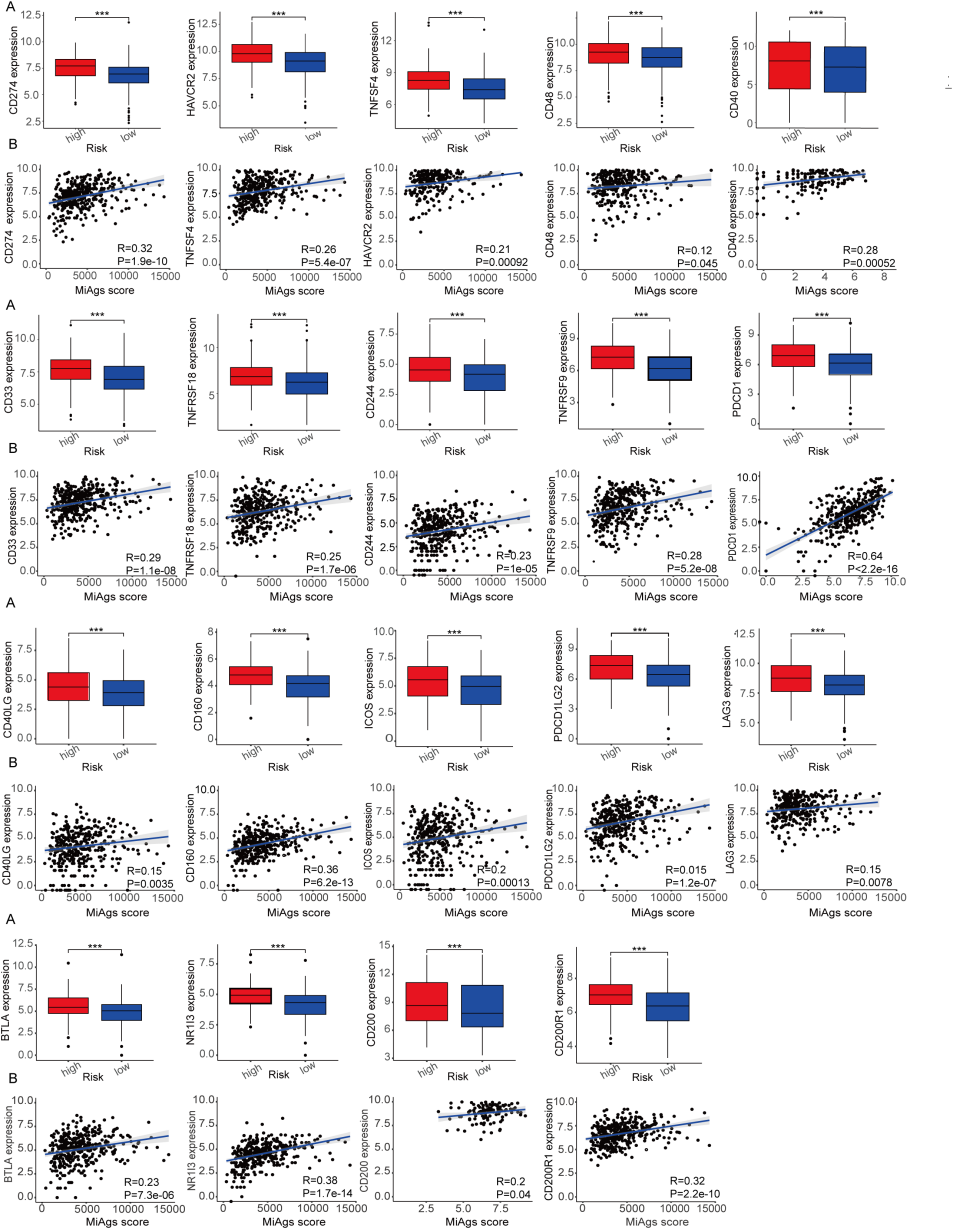
Correlation between MiAg score and immune checkpoints. **(A)** Expression of immune checkpoints between high and low risk groups for MiAg score. **(B)** Correlation between MiAg score and immune checkpoints. (p-values are shown as: ***P < 0.001).

We analyzed the expression levels of 19 common immune checkpoints and their correlations in the high and low MiAg score subgroups. As illustrated in **Figure 9A**, all 19 immune checkpoints were expressed at significantly higher levels in the high-risk group than in the low-risk group (P<0.001). These findings suggest that the high-risk group may have a more immunosuppressive microenvironment, which could contributing to disease progression and poorer outcomes. In addition, **Figure 9B** demonstrates that the 19 immune checkpoints showed a significant positive correlation with the MiAg score (P<0.05). These correlation indicates that higher MiAg scores, indicative of a more immunosuppressive environment, are linked with elevated expression of these immune checkpoints. The concurrent elevated expression of multiple immune checkpoints in the high-risk group, together with their positive correlation with the MiAg score, underscores the potential role of these checkpoints in promoting an immunosuppressive tumor microenvironment in ovarian cancer. Such an environment may contribute to increased disease aggressiveness and resistance to immunotherapy, highlighting the importance of targeting these pathways in treatment strategies for high-risk patients. Beyond the 19 immune checkpoints depicted in **Figure 9**, we also investigated the expression of eight other common immune checkpoints in the MiAg subgroups, the results of which are presented in the Appendix.

### 3.9 Chemotherapy drug sensitivity analysis and CCLE database

**Figure 10:**
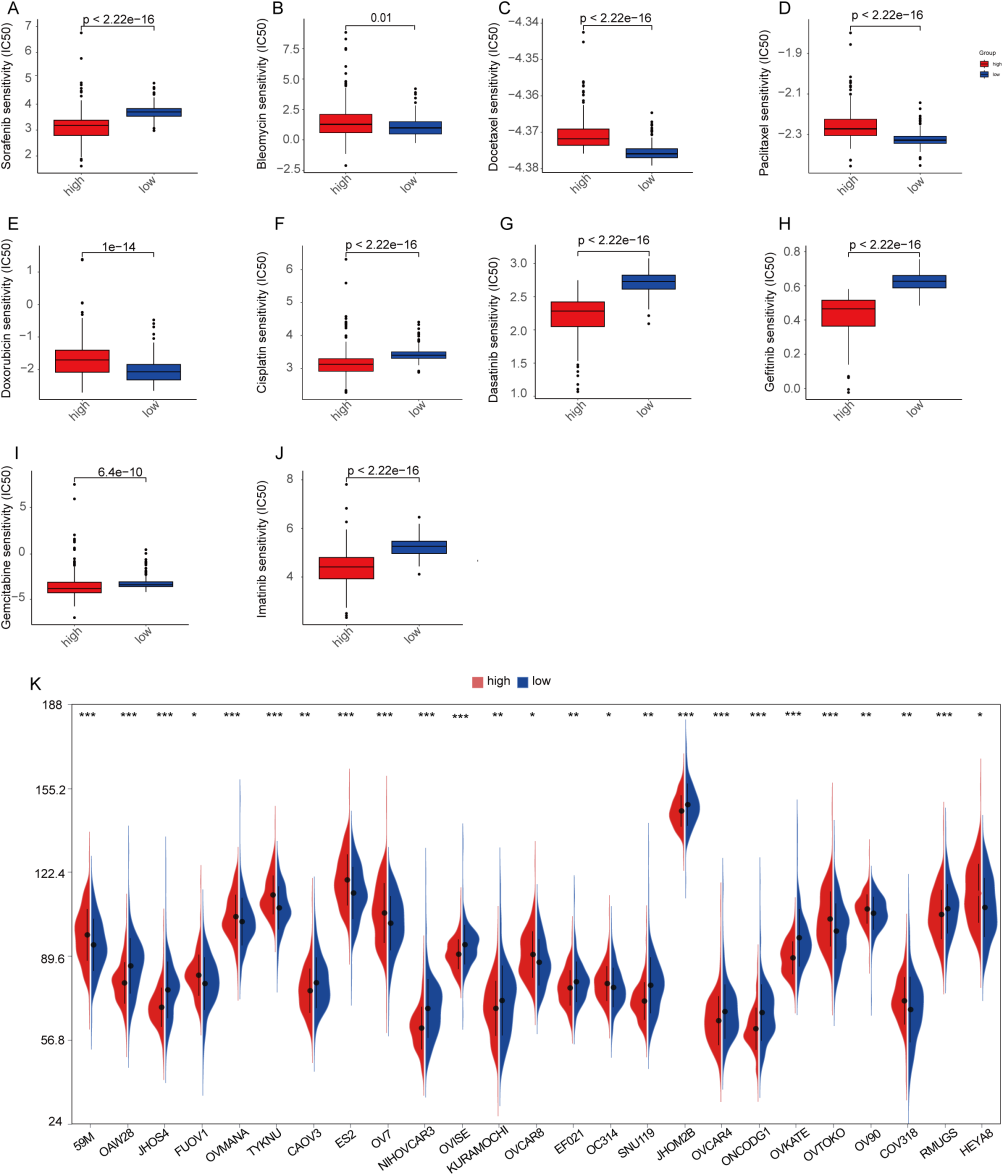
Chemotherapy drug sensitivity analysis and CCLE database. **(A–J)** Sensitivities of 10 ovarian cancer chemotherapeutic agents in high-risk and low-risk groups; **(K)** Expression of common ovarian cancer cell lines in high-risk and low-risk groups. (p-values are shown as: ns, not significant. *P < 0.05; **P < 0.01; ***P < 0.001).

Chemotherapy is the first-line treatment for patients with advanced ovarian cancer, and increasing rates of recurrence and treatment failure are closely associated with the development of drug resistance. Therefore, we compared the IC50 distributions of 10 commonly used chemotherapeutic agents between the high-risk and low-risk groups using the R package and assessed the sensitivity of both groups to these drugs. Additionally, we investigated whether MiAg could serve as a predictor of patient response to these chemotherapeutic agents. In this study, we selected 10 OC chemotherapeutic agents frequently used in clinical practice, including bleomycin, cisplatin, gemcitabine, paclitaxel, sorafenib, dasatinib, gefitinib, vincristine, veriparib, and imatinib. Our analysis revealed that the high-risk group was significantly less sensitive to bleomycin, docetaxel, paclitaxel, and doxorubicin, but exhibited greater sensitivity to sorafenib, cisplatin, dasatinib, gefitinib, gemcitabine, and imatinib (**Figures 10A-J**). These findings demonstrate that MiAg is a valuable tool for predicting the choice and sensitivity of chemotherapeutic agents in OC patients.

To ensure that subsequent *in vitro* cellular experiments could be conducted effectively, we analyzed the gene expression data of ovarian cancer cell lines from the CCLE database by examining differences between high- and low-MiAgs score subgroups. The results showed that 25 cell lines exhibited significant differences in gene expression between the two groups (**Figure 10K**).

### 3.10 HPA database

**Figure 11:**
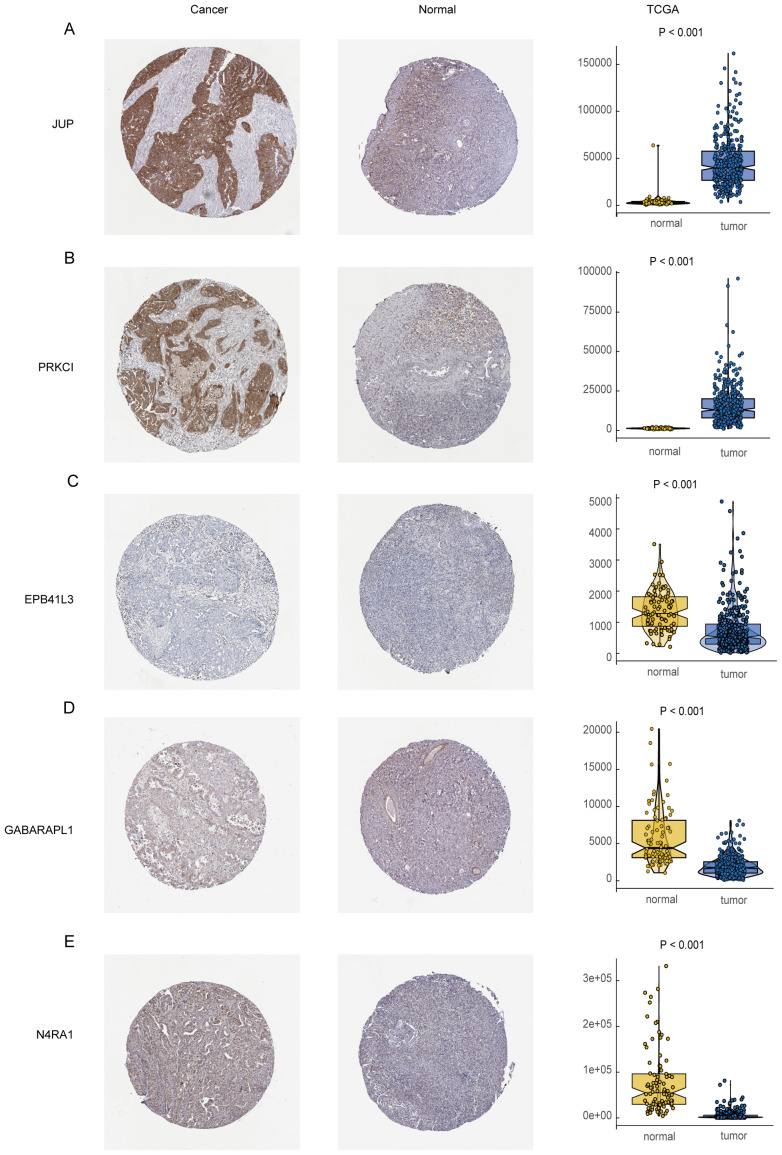
Immunohistochemical analysis of the HPA data base. **(A)** JUP expression in normal and ovarian cancer tissues; **(B)** PRKCI expression in normal and ovarian cancer tissues; **(C)** EPB41L3 expression in normal and ovarian cancer tissues; **(D)** GABARAPL1 expression in normal and ovarian cancer tissues; **(E)** N4RA1 expression in normal and ovarian cancer tissues.

We utilized immunohistochemical images sourced from the Human Protein Atlas database (HPA) to evaluate the protein expression levels of the five aforementioned prognostic genes. Our analysis compared protein expression in normal ovarian tissues and ovarian cancer tissues to identify potential differences (**Figure 11**). The findings revealed that JUP and PRKCI exhibited significantly elevated protein expression in OC tissues compared to normal ovarian tissues (**Figures 11A, B**). EPB41L3 expression was not detected in OC tissues but was observed at intermediate staining intensity in follicular cells of normal ovarian tissues, with no expression in ovarian stromal cells (**Figure 11C**). GABARAPL1 was primarily localized in the cytoplasm and cell membrane of OC tissues, predominantly showing low staining intensity, while its expression was undetectable in normal ovarian tissues (**Figure 11D**). N4RA1 demonstrated predominant nuclear expression with moderate staining intensity in OC tissues, whereas in normal ovarian tissues, follicular cells displayed moderate staining intensity, and ovarian stromal cells exhibited lower levels of staining (**Figure 11E**).

Additionally, we investigated the expression levels of these five prognostic genes in OC versus normal ovarian tissues using data from the TCGA cohort. The results corroborated the HPA database findings, confirming that JUP and PRKCI were expressed at higher levels in OC tissues than in normal tissues, whereas EPB41L3 was expressed at lower levels in OC tissues. However, the expression levels of GABARAPL1 and NR4A1 were lower in OC tissues compared to normal tissues, contradicting the HPA database observations. This discrepancy may stem from the relatively small sample size of the HPA database, highlighting the need for further validation studies to confirm these findings.

## 4 Discussion

Cancer treatment is advancing, with HER2-targeted therapy, glycolysis-targeted therapy, and choline metabolism-targeted therapy showing promise (28, 29). However, clinical application faces challenges such as drug resistance, safety issues, metabolic heterogeneity, and a complex immune microenvironment. Further research is needed to optimize treatment regimens and explore combination strategies to improve efficacy and patient prognosis. Ovarian cancer, a highly aggressive malignancy in women, urgently requires new therapeutic approaches. Mitochondrial autophagy, which removes damaged mitochondria, and cellular senescence, a hallmark of aging, are both closely linked to ovarian cancer development and may offer potential therapeutic avenues (12, 13). On one hand, mitochondrial autophagy is integral to drug resistance, tumor cell evolution, and the tumor microenvironment in ovarian cancer. On the other hand, mounting evidence supports a strong association between mitochondrial autophagy and aging processes. Thus, these two mechanisms are intricately interconnected in both the development and treatment of ovarian cancer. However, the specific genes governing this interplay remain largely unexplored in this context.

The interaction between MiAg-related genes and OC progression, as well as their association with patient survival, remains largely unknown. In our study, we identified five key MiAg-associated genes—JUP, NR4A1, GABARAPL1, PRKCI, and EPB41L3—that may play significant roles in OC pathogenesis. JUP (plakoglobin) is elevated in early-stage ovarian cancer and has been proposed as a potential biomarker (30, 31). Although JUP has been described as a tumor suppressor, inhibiting OC cell growth, migration, and invasion (31), its precise role remains under investigation. NR4A1, a member of the NR4A nuclear receptor family, is implicated in carcinogenesis, apoptosis, inflammation, and metastasis. Notably, NR4A1 exhibits a dual role in cancer, it can act as either a tumor suppressor or promoter, depending on the cellular context, particularly via the TGF-β signaling pathway (32). In high-grade OC, elevated NR4A1 levels have been linked to poorer prognosis and increased metastasis, partly through the induction of EGR3 expression (33). However, conflicting evidence suggests that NR4A1 downregulation may be associated with chemosensitivity in OC, highlighting the need for further investigation (34). Our study suggests that NR4A1 functions as a potential risk factor in OC. GABARAPL1, a member of the ATG8 protein family, plays a crucial role in autophagy and has been identified as a tumor suppressor in several cancers (35). However, in OC, higher GABARAPL1 expression correlates with poorer patient outcomes, suggesting a complex and context-dependent role in tumor progression (36). PRKCI overexpression is an early event in OC development, as demonstrated by TCGA data and mouse models (37). It is one of the most frequently amplified and overexpressed genes in OC, driving cancer cell proliferation, migration, and invasion *in vitro*, as well as tumor growth *in vivo* (38). Elevated PRKCI expression has been particularly associated with enhanced proliferative and metastatic potential in clear cell OC, supporting PRKCI as a promising therapeutic target (39). EPB41L3, known for its tumor-suppressive properties, is frequently downregulated in OC compared to normal tissues (40). In epithelial OC, EPB41L3 expression is often absent or reduced due to methylation, which impairs its tumor-suppressive function and promotes cancer progression (41). Despite increasing evidence linking mitochondrial autophagy and cellular senescence to OC, the role of MiAg-related genes in this interplay remains underexplored. Most existing studies have examined these processes separately. Our analysis integrates mitochondrial autophagy and cellular senescence genes, providing a comprehensive perspective on their collective impact on OC development and prognosis.

To date, the effects of mitochondrial autophagy and cellular senescence genes in ovarian cancer remain insufficiently explored. In our study, we developed a scoring system called the MiAgscore to assess mitochondrial autophagy and cellular senescence in OC patients. We first examined the expression levels of 560 MiAg-related genes in both OC and normal tissues. Among these, 52 genes were identified as differentially expressed. However, when we performed consistent cluster analysis based on these DEGs, the resulting two subgroups did not show significant differences in clinical characteristics (P = 0.76). To further evaluate the prognostic significance of these MiAg-associated modifiers, we constructed a prognostic model featuring a 5-gene risk profile using Cox regression analysis and LASSO regression analysis. Based on the median risk score, TCGA samples were categorized into high-risk and low-risk groups to assess the survival impact of these subgroups. The results demonstrated that patients in the high-risk group had poorer survival outcomes, while those in the low-risk group exhibited better survival outcomes. Similar findings were confirmed through validation in the GEO dataset. To improve the clinical applicability of our study, we conducted univariate and one-way COX analyses, and developed a nomogram with calibration curves for further validation. Additionally, we performed functional enrichment analysis of DEGs between the low-risk and high-risk groups to explore the underlying biological mechanisms.

Tumor infiltrating immune cells are closely correlate with immunotherapy efficacy and cancer patient survival (42, 43). Our analysis revealed that the low-risk group exhibited higher infiltration of immune cells associated with antitumor responses, including activated CD8^+^ T cells, effector memory CD8^+^ T cells, neutrophils, and memory B cells. Conversely, the high-risk group displayed increased infiltration of immunosuppressive cells, such as Tregs and pDCs, suggesting a more immunosuppressive environment and potentially poorer prognosis. We conducted analyses of 16 immune cell infiltrations and 13 immune profiles, which underscored the significance of immune cell patterns in predicting patient prognosis and guiding personalized immunotherapy strategies. Notably, the high-risk group may exhibit a distinct immune profile characterized by elevated levels of multiple immune signatures, implying a more complex or active immune environment in these patients. These findings emphasize the critical role of immune signature profiles in the risk stratification of OC patients and the development of personalized treatment strategies. Future research should aim to validate these immune profiles in larger cohorts and investigate their potential as therapeutic targets to improve the prognosis of OC patients.

Chemotherapy remains a mainstay treatment for ovarian cancer. We determined the IC50 values of commonly used chemotherapeutic agents and compared their effects between high-risk and low-risk groups. The results showed that patients in the low MiAg score group were more sensitive to bleomycin, docetaxel, paclitaxel, and doxorubicin, whereas those in the high MiAg score group exhibited greater sensitivity to sorafenib, cisplatin, dasatinib, gefitinib, gemcitabine, and imatinib. These findings indicate that risk scores may serve as a useful tool for predicting chemotherapeutic response in OC patients.

In summary, our study demonstrated that mitochondrial autophagy and cellular senescence are closely associated with the onset and progression of OC. We developed a novel MiAg score, which offers a genetic signature for predicting patient prognosis. We further performed functional, immunological, mutational, drug sensitivity, and cell lineage analyses to confirm the reliability and clinical applicability of the MiAg score. This scoring system has the potential to predict the effectiveness of adjuvant chemotherapy and immunotherapy, providing clinicians with valuable insights for treatment planning. However, our study has certain limitations. Potential bias may arise from the limited clinical data available in public datasets. Thus, future clinical trials are warranted to further validate our findings and assess the broader clinical utility of the MiAg score.

## Data Availability

The datasets presented in this study can be found in online repositories. The names of the repository/repositories and accession number(s) can be found below: GSE53963.
