# Supplementary figures and images for: A novel mitochondrial autophagy and aging-related gene signature for predicting ovarian cancer
Source: Front Immunol. 2025 Jun 5;16:1594021. doi: 10.3389/fimmu.2025.1594021 (PMC12177532; doi:10.3389/fimmu.2025.1594021)

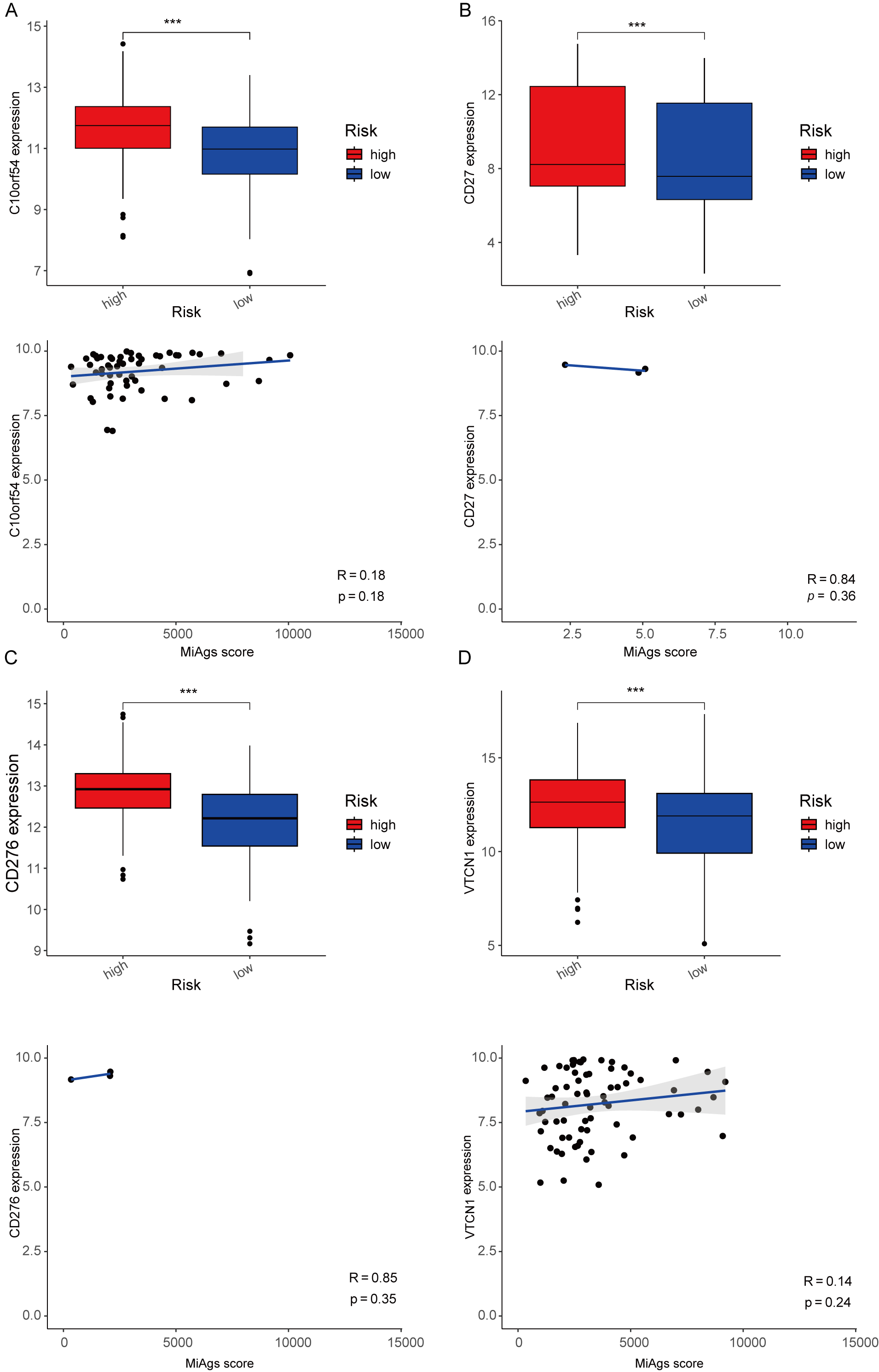

Supplement: Supplementary file 1 [file Image1.tif]

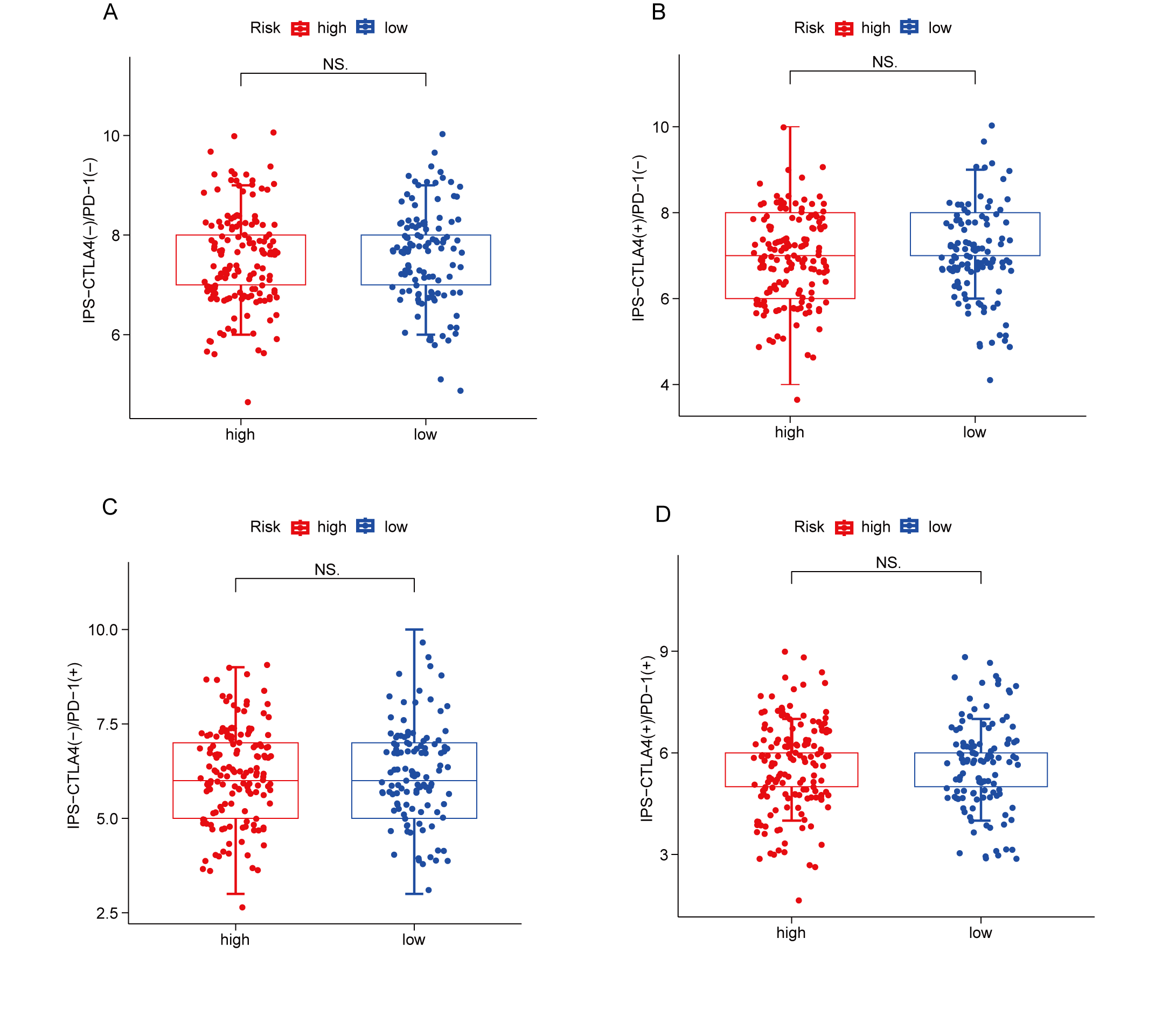

Supplement: Supplementary file 2 [file Image2.tif]
